# Divergent effects of straw and biochar single additions on soil water-heat-salt transport and corn productivity in arid salinized irrigation area

**DOI:** 10.3389/fpls.2025.1503943

**Published:** 2025-04-25

**Authors:** Wei Yang, Xiaomin Zhang, Yibo Zhao, Dongliang Zhang, Junjie Li, Riquan Song, Liping Wang, Zhongyi Qu

**Affiliations:** ^1^ College of Grassland Science, Inner Mongolia Agricultural University, Hohhot, China; ^2^ Key Laboratory of Grassland Resources of Ministry of Education, Inner Mongolia Agricultural University, Hohhot, China; ^3^ Autonomous Region Collaborative Innovation Center for Integrated Management of Water Resources and Water Environment in the Inner Mongolia Reaches of the Yellow River, Inner Mongolia Agricultural University, Hohhot, China; ^4^ College of Water Conservancy and Civil Engineering, Inner Mongolia Agricultural University, Hohhot, China; ^5^ Water Resources Research Institute of Inner Mongolia Autonomous, Hohhot, China; ^6^ College of Energy and Environment, Inner Mongolia University of Science and Technology, Baotou, China

**Keywords:** salinization, straw application mode, soil physical properties, soil moisture, corn, crop yield, water use efficiency

## Abstract

Straw return and straw-derived biochar are promising practices for improving soil physicochemical properties and crop production. However, the integrated effects of a single application on soil moisture, heat, salinity transport, and their regulation mechanism on crop water use efficiency (WUE) in salt-affected soils are still understood deeply. Four amendments were used: control without any additives (CK), direct return of 10 t ha^-1^ straw (BJ), and biochar treatments of 15 t ha^-1^ (B15), and 30 t ha^-1^ (B30). Application of straw and biochar generally increased the soil moisture content during whole crop growth periods. Temperature in the top 10 cm of soil increased by 0.97°Cfor B30 and 1.08°C for BJ when averaged two growing seasons. The BJ led to a slight reduction in soil pH from 0~30 cm, while biochar application did not significantly increased soil pH during crop growth periods. B30 also did not increased soil salinity of top 30-cm depth while BJ increased soil salinity. The desalting ratio at 0~30 cm at maturity in BJ and B30 two amendments decreased slightly during the first growing season but increased during the second growing season across two years. Straw and biochar also enhanced crop yield, WUE, net income. These effects improved more in the first year than in the second year. The two-year average WUE and net profit values increased more for B30 than for BJ. Thus, B30 amendment is recommended to improve soil water-heat environment, crop WUE, and net income without significantly adjusting the degree of soil salinization.

## 1 Introduction

Soil salinization is a major constraint in ensuring food security and improving the ecological environment (Hou et al., 2022; Sheoran et al., 2021). Soil salinization leads to extensive soil degradation and reduced crop productivity in semiarid and arid areas globally (Zhang et al., 2023a). Approximately 6.5% of the world’s arable and marginal soils are sodic or saline (Sheoran, 2021). Salt-affected lands are difficult to manage and often lead to abandonment. The poor physical structure and low crop productivity of saline soils have considerable adverse effects on crop morphological and physiological growth (Khasanov et al., 2023; Wang et al., 2023a), seriously limiting the sustainable development and comprehensive utilization of farmland soil (Singh et al., 2021).

Water and temperature are crucial regulators of microbial activities and biochemical cycles of carbon and nitrogen in the topsoil layer (Acharya et al., 2024; Zhang et al., 2024). The water and temperature dynamics influence soil characteristics and the highly efficient use of irrigation water and fertilizer resources in saline farmlands. These regulators are also critical variables in process-based agroecosystem models. Thus, field assessment is important for accurately simulating farmland hydrological processes, carbon and nitrogen cycles, nutrient uptake by plants, and crop growth and development in the soil and plant atmospheric continuum (Qi et al., 2020; Zhang et al., 2020). Additionally, the transport of salts in soils is mainly affected by water and temperature, these three soil environmental factors could further regulate soil quality and crop productivity. Studies of water-heat-salt transport dynamics and decreases in soil salts with agronomic practices are key to improving and preventing soil salinity in regions with water deficits (Yuan et al., 2023; Zhang et al., 2023b).

The average harvested residue output in China between 2018 and 2020 was 60 million tons, with a total crop straw production of 740 million tons in 2020 (Zhao et al., 2024). Problems with centralized straw collection and storage, large differences in straw texture, and insufficient recycling technology for active straw components have limited the comprehensive utilization of straw in China (Chen et al., 2020; Han et al., 2024). Straw- and straw-derived biochars returned to farmland are widely recognized as soil amendments owing to their special nature (Hou et al., 2020; Singh et al., 2021; Das et al., 2022; Hu et al., 2024). Recent studies have reported their crucial roles in regulating soil microbes (Dahlawi et al., 2018; Yang et al., 2022; Wang et al., 2023b), soil water-heat-salt properties (Cao et al., 2021; Liu et al., 2021; Ding et al., 2023), crop growth and yield (Hu et al., 2021; Yang et al., 2024), and water fertilizer productivity (Abbas et al., 2020; Yakubu et al., 2020). The application of 30 t ha^-1^ biochar reportedly enhanced the average soil temperature by 2°C, reduced day-night temperature differences, and increased the average maize leaf temperature by 2.2°C. The result was an increased corn yield of 11.9% in weakly alkaline farmland soils in arid and semiarid areas (Feng et al., 2021). In tropical Acrisols, biochar significantly improved soil water content and moderated soil temperature under conservation tillage (Obia et al., 2020). Ding et al. (2019) showed that soil temperature was more sensitive to changes in external (air and water) temperatures after biochar application to saline soil. Application of straw biochar can exacerbate salinization and change the soil salt distribution, enabling desalinization with little water consumption (Cong et al., 2023; Lee et al., 2022). The addition of halophyte biochar reportedly resulted in significant increases in soil water and salt content, and the application of 4% plant-derived biochar maintained more soil moisture content than soil salinity (Dong et al., 2022). Zhang et al. (2020) demonstrated that all biochar applications modes did not significantly affect soil moisture content in the top 20 cm soil layer, with no effect on the general trend of water and heat change in the soil tillage layer. However, the comparative effects of a single application of straw or biochar on soil water-heat-salt transport and the correlation with crop water use efficiency (WUE) under water-saving irrigation in the saline farmland of arid regions are unknown.

The Hetao Irrigation District located in the arid region of Inner Mongolia is an important grain-producing area in Northwest China. The region features few water resources, uneven temporal and spatial distribution of rainfall, and severe secondary soil salinization (Ding et al., 2019). As affected by the time of water diversion and distribution from the Yellow River, the irrigation time is often seriously disconnected from the critical period of crop water demand, and crop yield reduction is prominent owing to water shortages and salinization (Cao et al., 2023). In addition, the soil structure of saline farmland is poor, with low organic carbon content and weak retention of water and fertilizer by the soil. These properties seriously limit the improvement of crop yield and water and fertilizer utilization efficiency (Feng et al., 2023b).

Because of the various climatic and soil texture conditions, any changes in soil water, heat, and salt may be inconsistent following the application of straw or straw biochar, and the combined soil water-heat-salt dynamics could jointly change crop growth-based soil environments, further influencing crop water productivity. With the assumption that straw or biochar can completely affect soil water-heat-salt changes in different ways and regulate crop growth and water use efficiency, the objectives of this study were to: (i) compare the variations in soil temperature, moisture, and salts during crop growth periods with the addition of straw or biochar under drip irrigation with mulching, (ii) determine the response mechanism of crop water WUE to combined water-heat-salt interactions, and (iii) propose reasonable crop straw application modes for saline soil improvement.

## 2 Materials and methods

### 2.1 Description of study site

**Figure 1:**
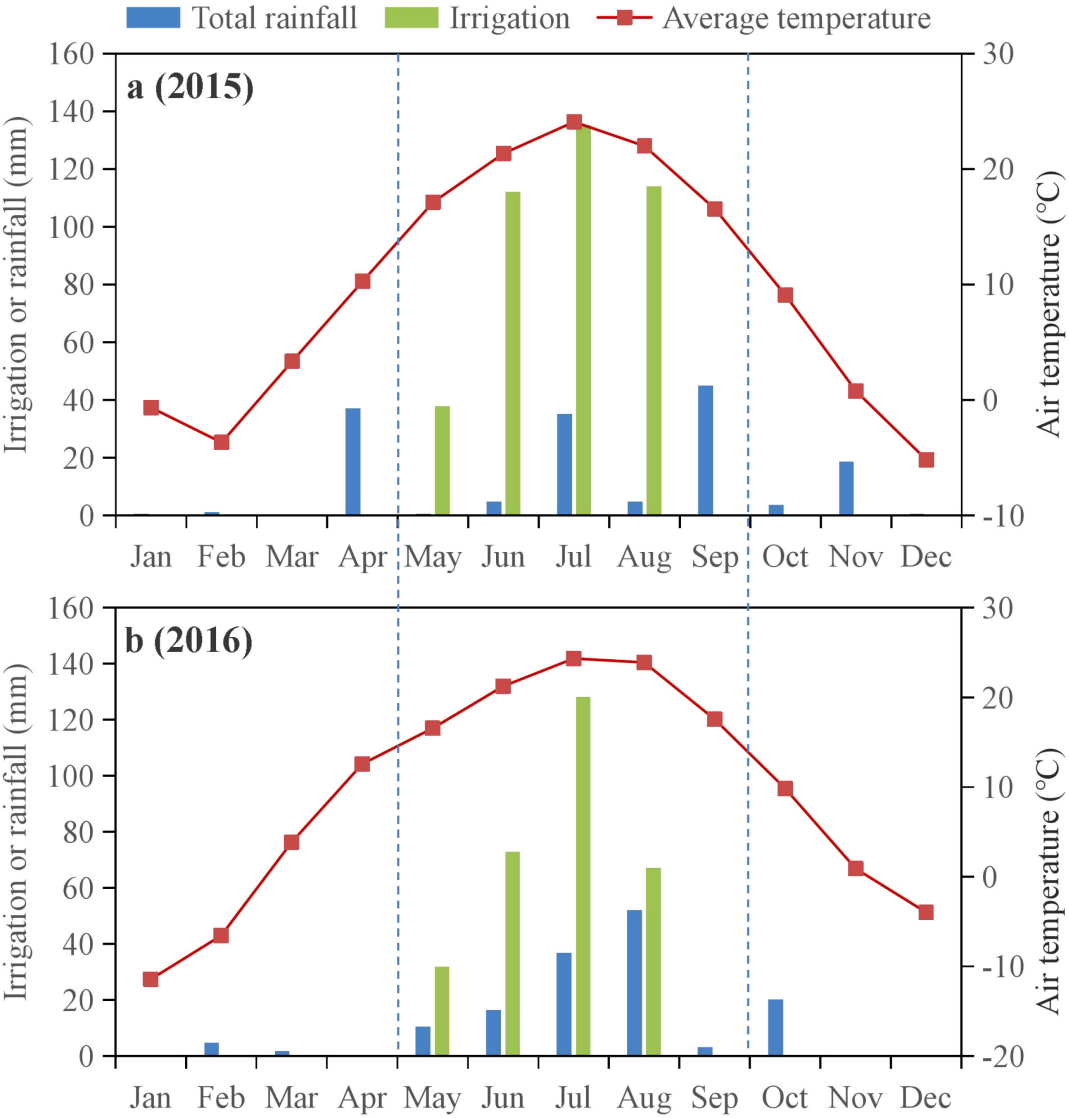
Air temperature and amounts of rainfall and irrigation during the 2015 **(a)** and 2016 **(b)** crop growth periods.

A field trial was performed in the 2015 and 2016 corn growing seasons at Jiuzhuang Experimental Station in Shuanghe Town, Linhe District, Bayannur City, Inner Mongolia, China (107°18′E, 40°41′N). The study site has an altitude of 1042 m, average temperature of 7.6°C, maximum temperature of 40.3 °C in July, and minimum air temperature of -35.3 °C in January. The frost-free period is approximately 130 days, with an average annual sunshine duration of approximately 3229 h. The annual accumulative rainfall is 137-214 mm, which is approximately 75% of the total rainfall from May through September (103-160 mm). The potential evaporation of the site of 1993-2373 mm is typical of an arid climate. During the 2015 and 2016 crop growth stages, the monthly average temperatures were 16.5-24.1 and 16.6-24.3°C, respectively, with total rainfall of 90 and 119 mm, respectively. The monthly total rainfall and mean monthly air temperature during the crop growth period are shown in **Figure 1**.

### 2.2 Experimental materials

The biochar used in this study was corn straw produced by the Liaoning Jinhefu Agricultural Development Co., Ltd. This product is made by burning corn straw at a carbonization temperature of 400°C for 8 h under anaerobic conditions. The main properties of the biochar are listed in **Table 1** (Yang et al., 2022). The straw was derived from corn straw harvested in the preceding year and cut into small segments of approximately 5 cm using a straw cutter.

**Table 1:** Physical and chemical properties of soil, biochar, and straw used in the study.

	Soil	Biochar	Straw
Texture	Sandy loam	NA	NA
Field capacity (cm^3^ cm^-3^)	0.25	NA	NA
Electric conductivity (μS cm^-1^)	318.5	NA	NA
pH	8.5	9.0	6.9
Organic matter (g kg^-1^)	14.5	925.7	1.5
Available P (mg kg^-1^)	5.3	394.2	2.7
Available K (mg kg^-1^)	184.0	783.9	28.1
Mass fraction of C (%)	33.4	47.2	43.1
Mass fraction of N (%)	1.47	0.7	0.6
Mass fraction of H (%)	0.068	3.8	5.4
C/N	22.7	67.4	71.8

NA, not available.

### 2.3 Experimental design

Four treatments were used. They were fully randomized and designed for the field experiment. The treatments comprised a blank control that did not apply biochar and straw (CK), direct return of 10 t ha^-1^ straw to the field (BJ), and biochar dosages of 15 and 30 t ha^-1^ (B15 and B30, respectively). Each treatment consisted of 12 individual plots with three replicates. Biochar and straw were applied to the soil surface using a rotary tiller to evenly mix with the topsoil at a mixing depth of approximately 15 cm.

Corn seeds (cultivar ‘Ximeng No.6’) were sowed into the experimental plots with a plant spacing of 30 cm and row spacing of 50 cm, with a planting density of 5,6667 plants ha^-1^. Each plot measured 90 m^2^ (15 m× 6 m). Prior to sowing, the base fertilizers for diamine phosphate (N:P_2_O_5_ = 14%:39%) and compound fertilizer (N:P_2_O_5_:K_2_O=30%:5%:5%) were 450 and 338 kg/ha, respectively, in both growing seasons. Additional nitrogen fertilizer (375 kg/ha) was provided as common urea via a drip irrigation system, with a total of 75 kg/ha each time for the entire growing season. Irrigation (22.5 mm) was done when the soil suction reached -25kPa. Irrigation was performed 17 times in 2015 and 13 times in 2016, with total respective irrigation amounts of 400 mm and 300 mm.

### 2.4 Sampling and measurements

#### 2.4.1 Soil physical and chemical properties

For the growth stages of corn plants (three-leaf, jointing, tasseling, grain-fill, and physiological maturity), soil samples from to 0-15, 15-30, 30-40, 40-60, and 60-80 cm were acquired. A portion of each sample was placed into aluminum boxes to measure soil moisture content using the oven-drying method. For each growth period, the soil temperature at the 10 cm layer was continuously measured using a model WNG-12 vertical angle thermograph from 6:00 am to 8:00 pm at 2-h intervals every day for three consecutive days. For each growing season, the average soil temperature for each observation period was calculated based on measurements taken at the same time for the three-day periods.

Soil samples taken from the 0-15 and 15-30 cm layers were dried under natural conditions and then passed through a 2-mm sieve to determine the electrical conductivity (EC) at a 1:5 soil:water ratio using a model DDS-307 portable electricity analyzer. Soil pH was determined using a model F2 acidometer. Soil salt content (SSC) for each soil layer was calculated (g/kg) as 6.9×EC (ds/m) -0.2. The soil desalinization ratio was defined as the ratio of the difference between the final SSC at harvesting and the initial SSC at sowing to the initial SSC.

#### 2.4.3 Crop yield and water use efficiency

Actual evapotranspiration (ET) for corn plants during the whole growth period each year was calculated using the Eqs. (1) (2) in a 0.8-m soil profile.


ET=ΔSWC+P+I+R+G−D

where, *ET* is the evapotranspiration, mm; *ΔSWC* is the difference in soil water storage in a 0.8-m soil profile between planting period and harvesting period, mm; *P* is the effective rainfall during growth period, mm; *I* is the irrigation amount, mm; *R* is surface runoff, mm. Drip irrigation has a smaller irrigation quota in this study, and the test area is flat, therefore there is no surface runoff. *G* is the amount of groundwater replenishment to crop roots during the growth period (mm). In this study, the root zone range was defined as 0 ~ 80 cm depth, and the depth of groundwater buried in the test area was greater than 1 m from the root layer, so groundwater recharge could be ignored (Zhou and Feng, 2020); *D* is the deep root zone leakage during the growth period, mm, which can be calculated according to the method provided in FAO 56, assuming rainfall or irrigation first recharge the root layer soil water to the field capacity, the excess water is the deep leakage loss (Chivenge et al., 2007). The calculation method is to add the effective soil moisture content and the irrigation amount (or precipitation) in the 80 cm soil layer before irrigation (or precipitation), and then subtract the field water capacity (Chivenge et al., 2007).

At physiological crop maturity, 20 plants from each replicate plot were quantified to determine the aboveground biomass and yield components. Crop aboveground tissues were dried in an oven at 105°C until stable weight to determine the total aboveground biomass. Corn WUE was calculated as the ratio of grain yield to the actual ET.

#### 2.4.4 Cost-benefit evaluation

The total cost was calculated as the sum of the experimental material and energy costs. The experimental materials included biochar, straw, plastic films, chemical fertilizers, corn seeds, pipelines, and pesticides. Energy included electricity and diesel oil related to rotary tillage, crop planting, biochar/straw soil application, and crop harvesting. The total income includes wood vinegar, tar, and gas generated during biochar production, and corn grain yield. The material, energy, and sorghum grain prices were based on market prices. Net income was estimated by subtracting the total costs from the gross profit (Uygan et al., 2021). The increased percentage of net profit was determined as the difference between the treatment and CK plots divided by the CK plot.

#### 2.4.5 Statistical analysis

For each sample, the measured values (*n* = 3) of each measurement between the four treatments were analyzed using one-way analysis of variance with SPSS (version 19.0; IBM, USA). The mean value for each parameter was compared at *p* < 0.05 the least significant difference test.

## 3 Results

### 3.1 Soil moisture dynamics

**Figure 2:**
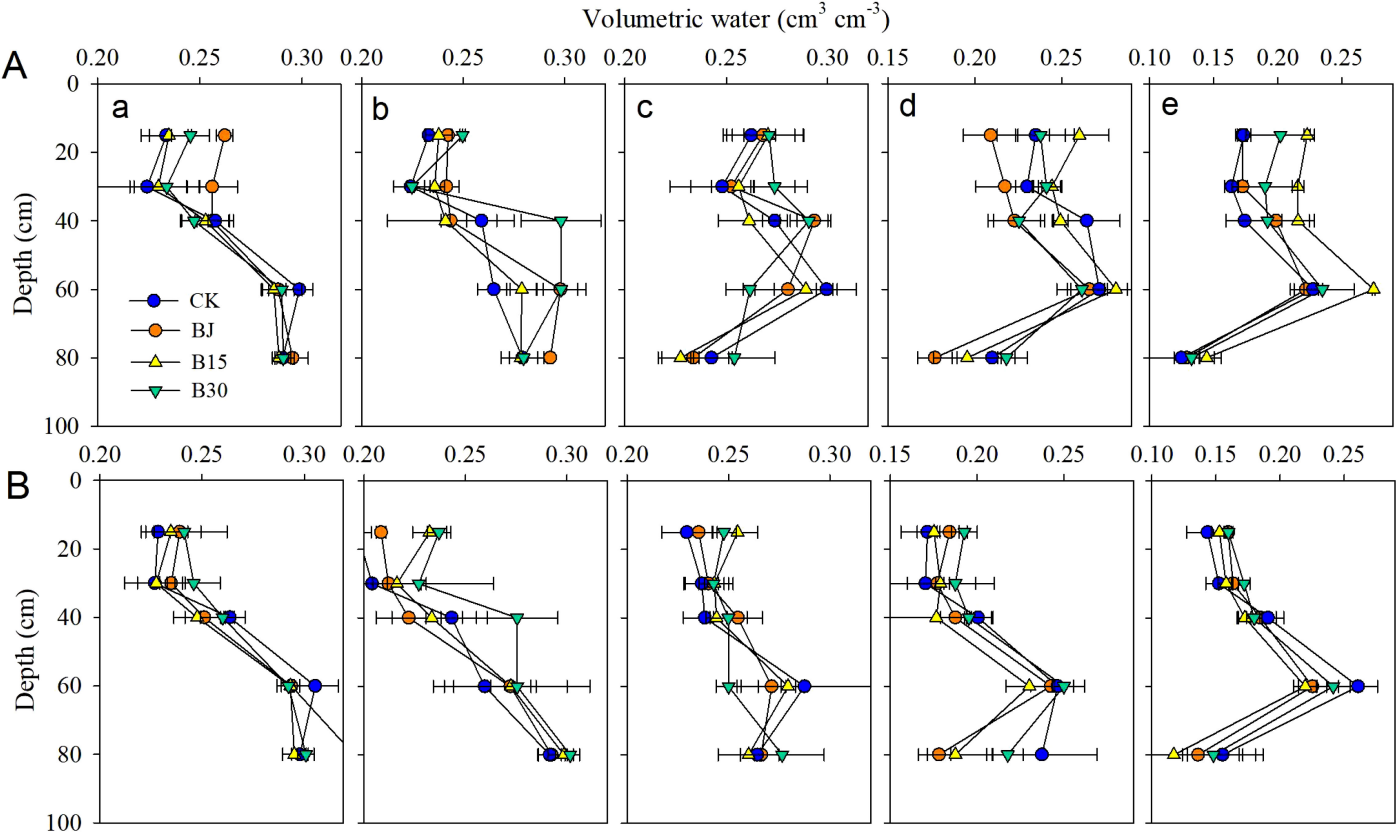
Volumetric water content at 0-15, 15-30, 30-40, 40-60, and 60-80 cm soil layers during (a) three-leaf, (b) jointing, (c) tasseling, (d) grain-fill, and (e) physiological maturitycm in 2015 **(A)** and 2016 **(B)**.

Soil moisture generally increased with increasing soil layer depth across the various biochar and straw treatments. Overall, the application of straw and straw-derived biochar increased the soil moisture content in the 0-80 cm soil zone in various growth periods of corn (**Figure 2**), especially in the 30 cm topsoil layer from the three-leaf to the tasseling growth period. The trend of change was basically the same for the two years of the study. Compared with the corresponding non-biochar control (CK), application of higher dosages of biochar (B30) and straw (BJ) significantly induced respective moisture content increases of 3.1% and 13.2% for three-leaf and 4.6% and 12.5% for jointing in 2015, and 4.1% and 5.4% for three-leaf and 4.9% and 7.5% for jointing in 2016. In contrast, the water content in soil deeper than 30 cm was not significantly different from that of biochar (B15 and B30), straw, and CK.

### 3.2 Soil temperature response

**Figure 3:**
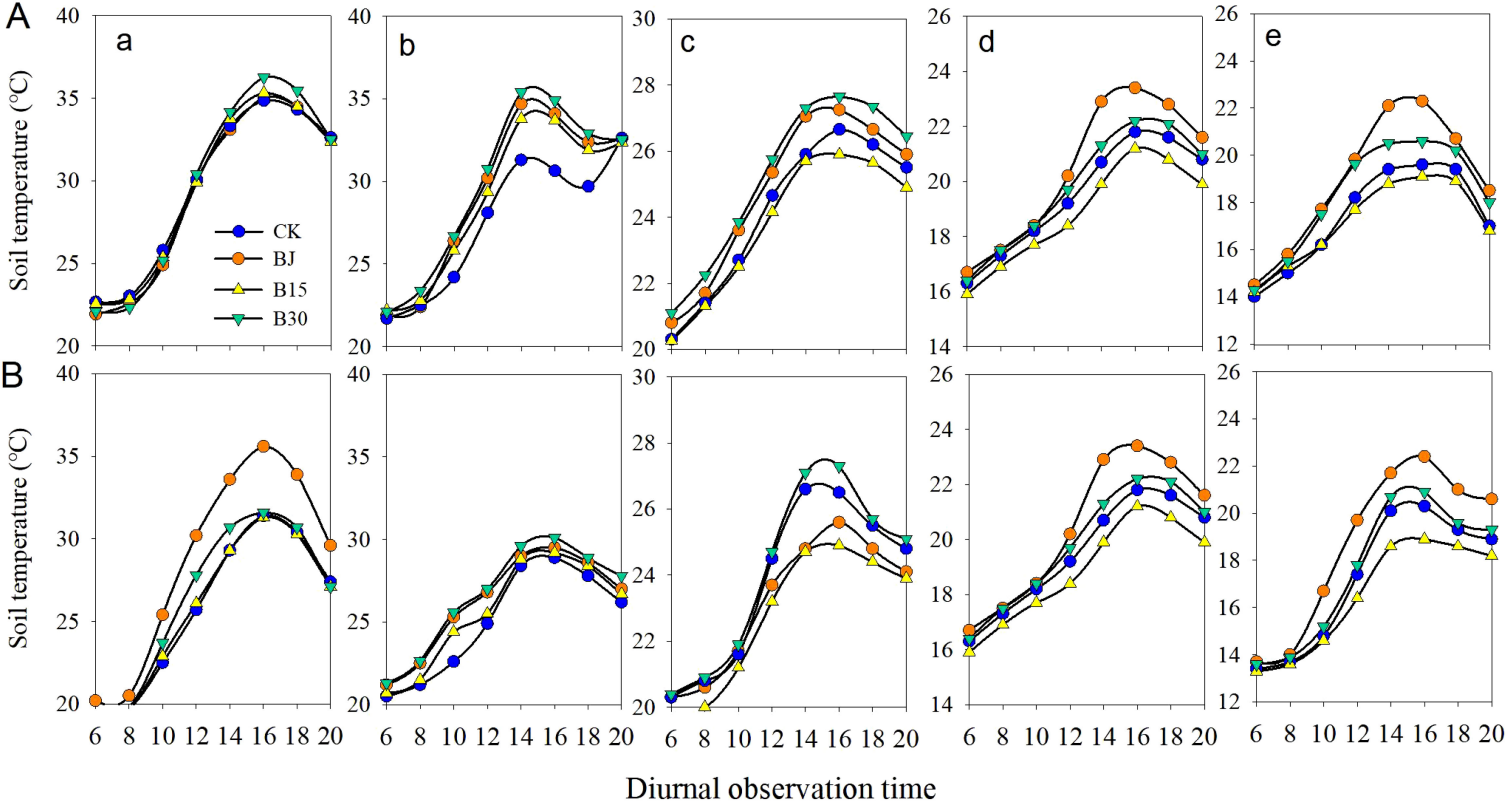
Soil temperature in the 0-10 cm soil layer from 6:00 to 18:00 at (a) three-leaf, (b) jointing, (c) tasseling, (d) grain-fill, and (e) physiological maturity in 2015 **(A)** and 2016 **(B)**.

For both the 2015 and 2016 growing seasons, compared with the non-biochar control plot (CK), treatments with biochar (B15 and B30) and straw (BJ) generally improved the soil temperature from 8:00 to 20:00 during the three-leaf and jointing growth periods (**Figure 3**; **Table 2**). From tasseling to physiological maturity, B30 and BJ induced increased soil temperature, whereas B15 resulted in decreased soil temperature. A lower dosage of biochar did not significantly increase soil temperature at the crop vegetative stage. Higher dosages of biochar and straw return applications (B30 and BJ) improved soil heat at the crop growing stage.

**Table 2:** Statistical results for soil temperature during crop growth periods between straw and biochar treatments.

Year	Growth periods	Treatment	Diurnal observation time							
			6:00	8:00	10:00	12:00	14:00	16:00	18:00	20:00
2015	Three-leaf	CK	a	a	a	a	a	b	a	a
		BJ	a	a	a	a	a	a	a	a
		B15	a	a	a	a	a	a	a	a
		B30	a	a	a	a	a	a	a	a
	Jointing	CK	a	a	a	a	b	b	b	a
		BJ	a	a	a	a	a	a	a	a
		B15	a	a	a	a	a	a	a	a
		B30	a	a	a	a	a	a	a	a
	Tasseling	CK	a	a	b	a	b	ab	a	ab
		BJ	a	a	a	a	a	a	a	ab
		B15	a	a	b	a	b	b	a	b
		B30	b	a	a	a	a	a	a	a
	Grain-fill	CK	a	a	a	a	ab	b	ab	a
		BJ	a	a	a	a	a	a	a	a
		B15	a	a	b	a	b	b	b	a
		B30	a	a	a	a	a	ab	a	a
	Maturity	CK	a	a	a	ab	b	b	b	a
		BJ	a	a	a	a	a	a	a	a
		B15	a	a	a	b	b	b	b	b
		B30	a	a	a	a	ab	ab	a	a
2016	Three-leaf	CK	a	a	a	b	b	b	b	a
		BJ	a	a	a	a	a	a	a	a
		B15	a	a	a	b	b	b	b	a
		B30	a	a	a	b	b	b	b	a
	Jointing	CK	a	a	b	b	b	b	b	a
		BJ	a	a	a	a	a	a	a	a
		B15	a	a	ab	a	ab	a	ab	a
		B30	a	a	a	a	a	a	a	a
	Tasseling	CK	a	a	a	a	a	a	a	a
		BJ	a	a	a	a	a	a	ab	a
		B15	a	a	a	a	b	a	b	a
		B30	a	a	a	a	a	a	a	a
	Grain-fill	CK	a	a	b	ab	b	b	a	a
		BJ	a	a	a	a	a	a	a	a
		B15	a	a	b	b	b	b	a	a
		B30	a	a	b	a	b	b	a	a
	Maturity	CK	a	a	b	b	b	a	ab	a
		BJ	a	a	a	a	a	a	a	a
		B15	a	a	ab	b	c	a	b	b
		B30	a	a	a	a	b	a	a	a

Different lowercase letters indicate significant differences between treatments for same sampling.

**Figure 4:**
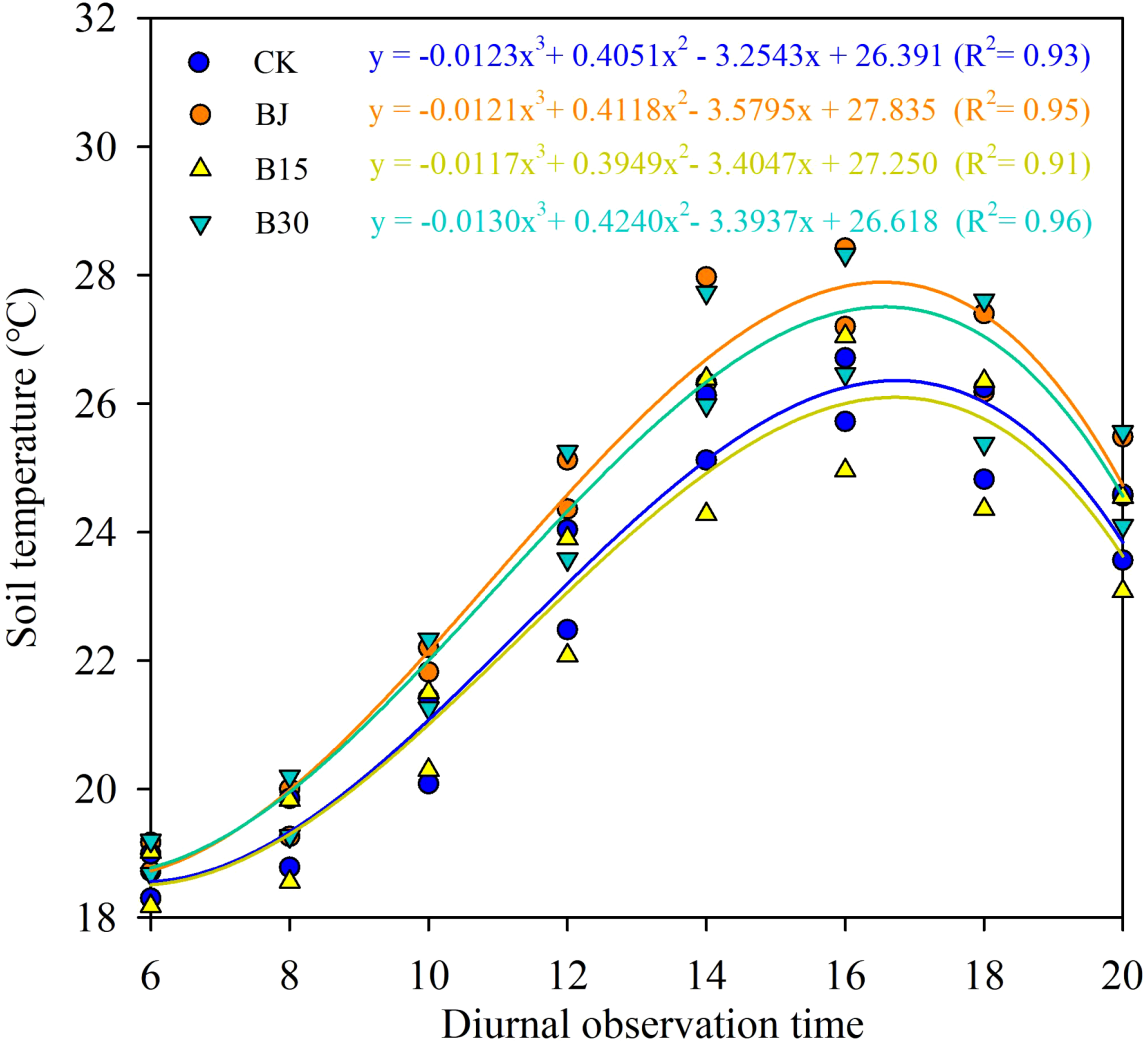
Nonlinear regressions between soil temperature and its measured time from 6:00-20:00 across growth periods over the 2 years of the study.

When these measured times from 6:00-20:00 in each growth period were averaged, compared with CK, B30 induced soil temperature increases of 0.22, 2.61, 1.05, 0.34, and 0.93°C (mean=1.03°C) at three-leaf, jointing, tasseling, grain-fill, and physiological maturity in the first growing season, respectively, and 0.65, 1.56, 0.31, 0.75, and 0.39°C (mean=0.73°C), respectively, in the second year. The mean values were 1.03 and 0.73 °Cfor whole growing stages in the first and second year, respectively. Similarity, for three-leaf, jointing, tasseling, grain-fill, and physiological maturity, BJ resulted in respective soil temperature increases of 1.97, 0.63, 0.95, and 1.58°C (mean=1.28°C) in the first year, and 1.18, 0.63, 1.01, and 1.49°C (mean=1.08°C) in the second year. Soil temperature increased by 0.97°C for B30 and 1.08°C for BJ when averaged across two growing seasons. The changes in soil temperature from 6:00 to 18:00 were also well demonstrated with nonlinear curves over 2 years (**Figure 4**).

**Figure 5:**
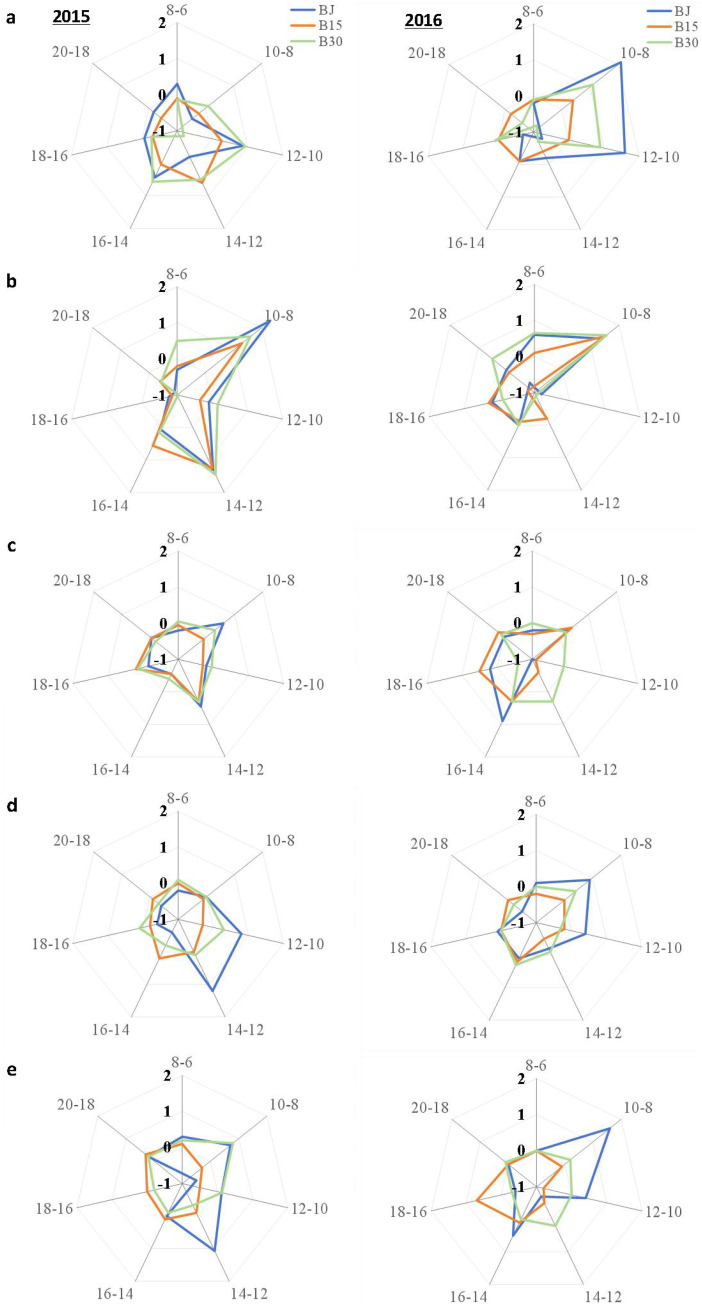
Net Increase d soil temperature induced by straw and biochar treatment, subtracting the control (CK) every 2 h from 6:00 to 20:00 within two measuring times (8:00-6:00, 10:00-8:00, 12:00-10:00, 14:00-12:00, 16:00-14:00, 18:00-16:00, 20:00-18:00) at **(a)** three-leaf, **(b)** jointing, **(c)** tasseling, **(d)** grain-fill, and **(e)** physiological maturity.

Further analysis showed that relative to CK (**Figure 5**), the increased soil temperatures between B30 and BJ within the two adjacent measurement times mainly occurred at 10:00-12:00, 8:00-10:00, 8:00-10:00, 12:00-14:00, and 12:00-14:00 at three-leaf, jointing, tasseling, grain-fill, and physiological maturity in the first year, and 8:00-10:00, 8:00-10:00, 14:00-16:00, 8:00-10:00, 8:00-10:00 in the second year, respectively.

### 3.3 Soil pH dynamics

**Figure 6:**
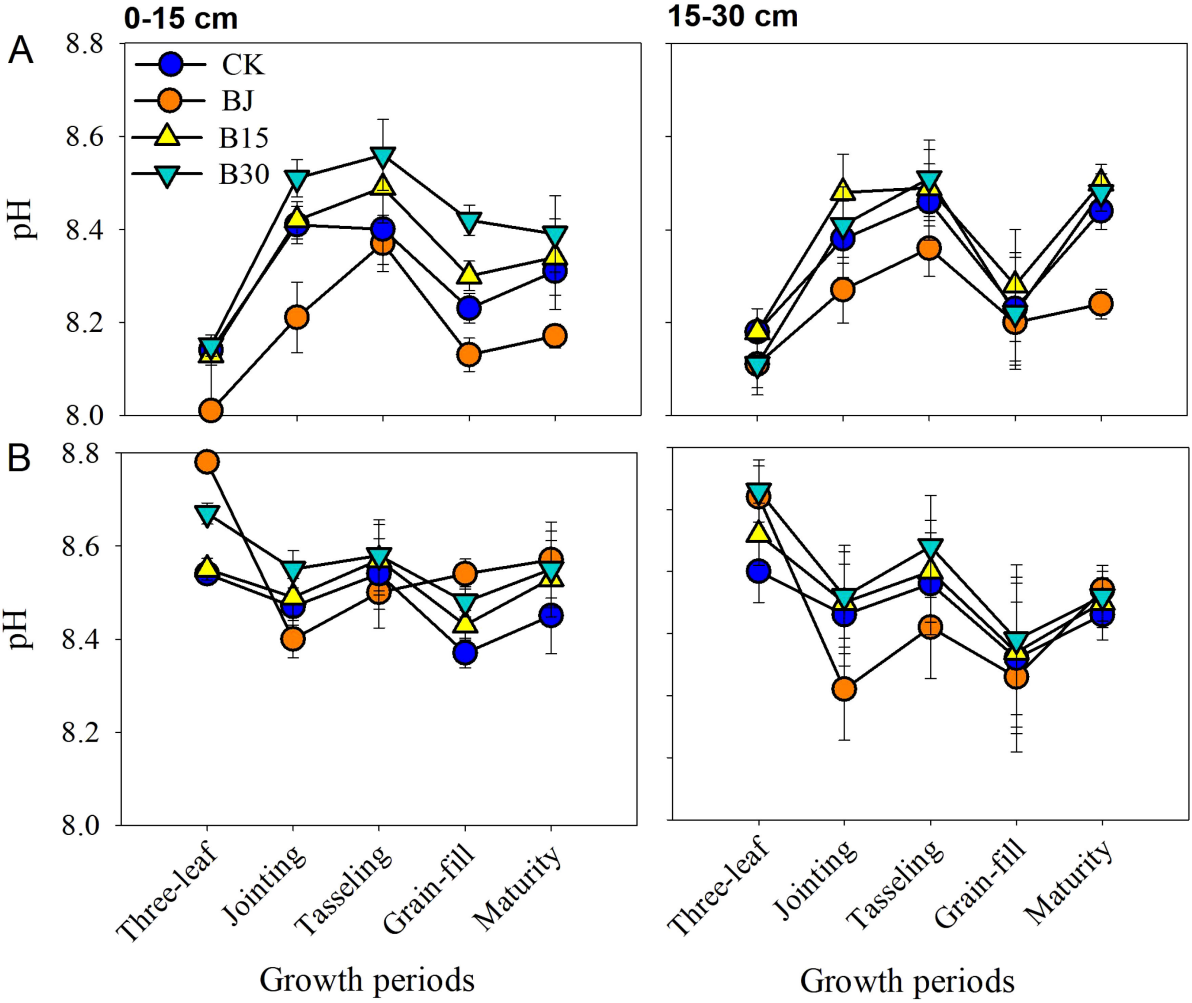
Soil pH in the 0-15 and15-30 cm soil layers during crop growth periods in 2015 **(A)** and 2016 **(B)**.

As shown in **Figure 6**, the pH values in the 0-15 and 15-30 cm soil layers initially increased and then decreased the entire 2015 growing season. In the 2016 growing season, these values displayed a “W” pattern of dynamics. The pH values were generally higher in B30 and B15 than in CK, and were lower in BJ than in CK when averaged across these five measurements. When averaged for the two years of the study, in 2015, the pH increased by 0.12 units for B30, but decreased by 0.09 units for BJ in the 0-15 cm soil layer, and increased by 0.04 units for B30 but decreased by 0.11 units for BJ in the 15-30 cm soil layer. In 2016, B30 significantly increased pH by 0.10 units in the 0-15 cm soil layer and by 0.14 units in the 15-30 cm soil layer. Increased pH was observed for BJ in the 0-15 cm soil layer, whereas B15 did not significantly improve pH.

### 3.4 Soil salt transport

**Figure 7:**
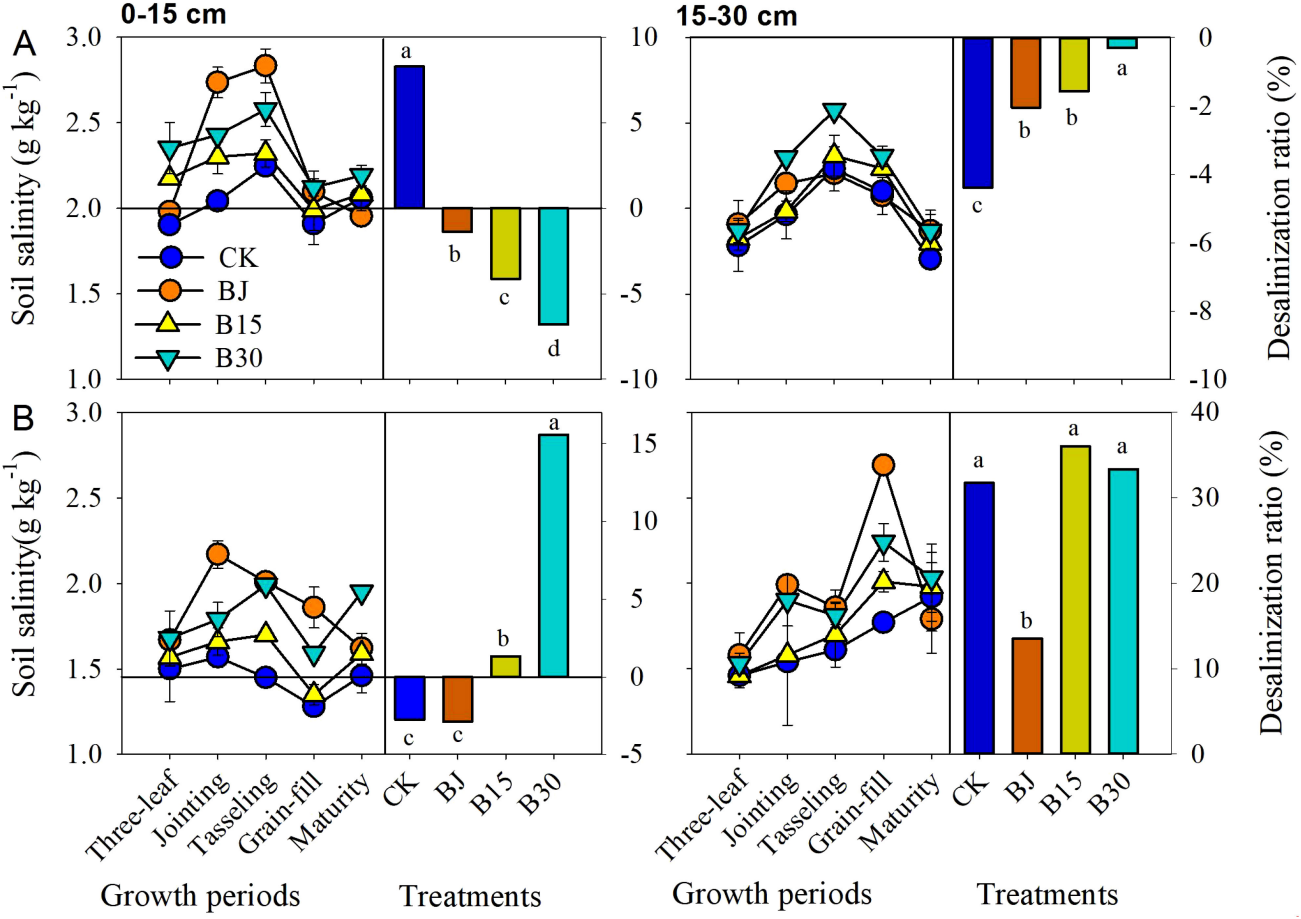
Soil salinity and desalting ratio in the 0-15 and 15-30 cm soil layers during crop growth periods in 2015 **(A)** and 2016 **(B)**.

Soil salts were <3.0 g kg^-1^ (1.28~2.83 g kg^-1^) in the 0-15 and 15-30 cm soil layers in each growing period (**Figure 7**). Generally, an increase before tasseling was followed by a decrease after tasseling in the 2015 and 2016 growing seasons. In all 0-15 and 15-30 cm soil layers, salts were higher in the biochar and straw treatments than in CK each year. When averaged across these sampling dates in 2015, the salt content in BJ, B15, and B30 was 14.2, 7.1, and 14.9% higher, respectively, than CK in the 0-15 cm soil layer, and 5.4, 4.8, and 12.7% higher, respectively, than CK in the 15-30 cm soil layer. Similarly, in 2016, the salt content in BJ, B15, and B30 was 28.4, 8.4, and 23.9% higher, respectively, than CK in the 0-15 cm soil layer, and 19.5, 5.2, and 18.2% higher, respectively, than CK in the 15-30 cm soil layer. These findings indicate that biochar application in the first year and straw return improved the salt content of the topsoil during the current and subsequent growing seasons, especially in the subsequent growing season.

### 3.5 Corn water productivity and net benefit

**Figure 8:**
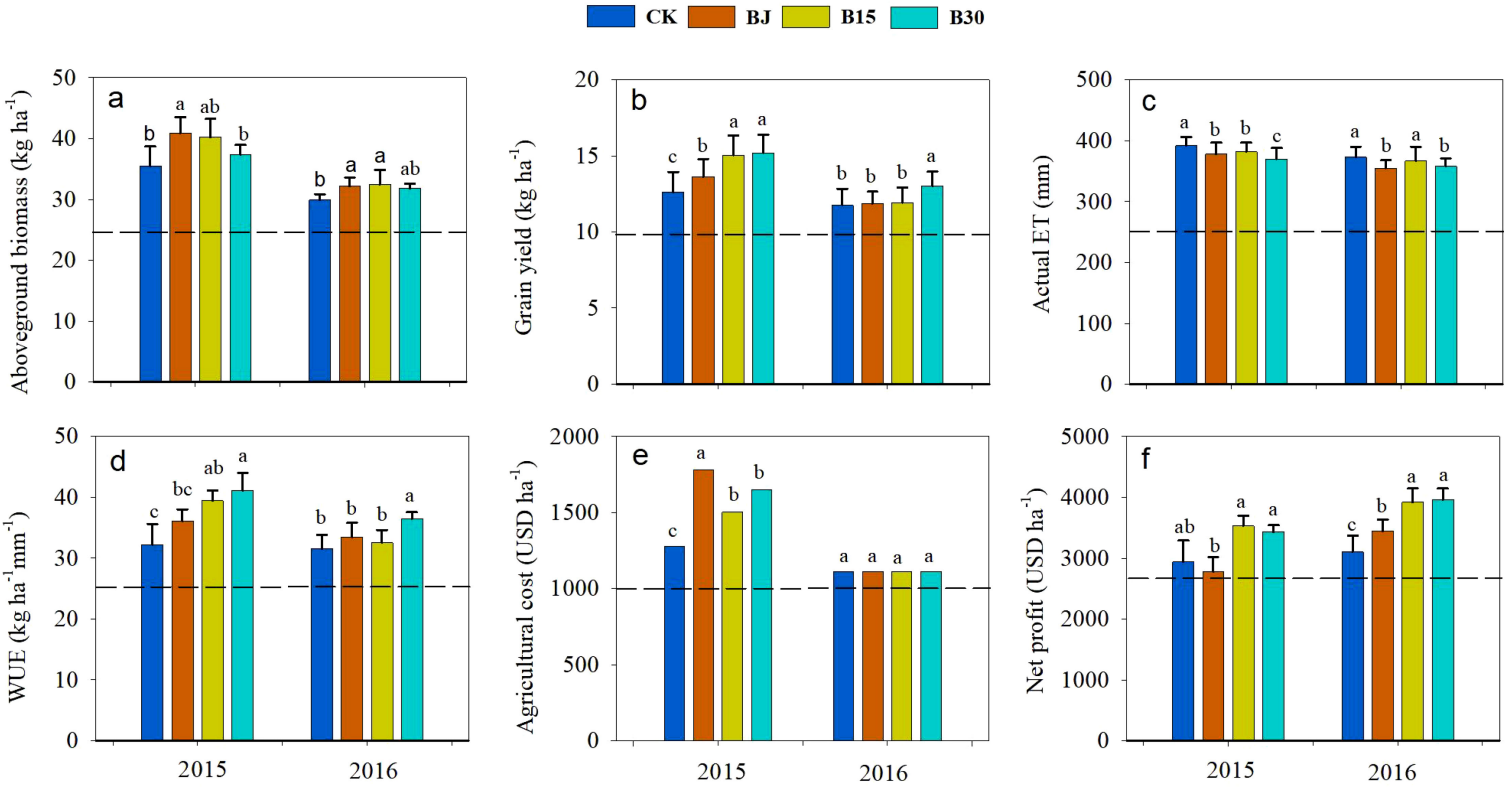
Aboveground biomass **(a)**, grain yield **(b)**, actual ET **(c)**, and water use efficiency (WUE, **d**), cost **(e)**, and net profit **(f)** for corn production under straw- and biochar-added saline farmland.

Straw and biochar generally promoted crop growth and net profit in terms of increased aboveground biomass, grain yield, and economic benefits compared to CK (**Figure 8**). Comparatively, the increases in yield and WUE were more pronounced in the first year than in the second. In 2015, aboveground biomass significantly improved by 15.2, 13.3, and 5.2% in BJ, B15, and B30, respectively, and grain yield significantly increased by 8.1, 19.3, and 20.4% in BJ, B15, and B30, respectively. In 2016, a significant increase in aboveground biomass occurred in B15 and BJ and a significant increase in yield occurred in B30. The actual ET showed a slight change each year, with reductions of 10-22 mm in 2015 and 10-15 mm in 2016 under the B15 and B30 treatments, and by 15 mm in 2015 and 18 mm in 2016 the BJ treatment. Thus, crop WUE was improved by straw and biochar treatments, as grain yield increased and ET decreased slightly. WUE increased by 12.1% for BJ, 22.4% for B15, and 27.6% for B30 in 2015. In 2016, the increase was significant for B30 and not for B15 or BJ. The net profit was also enhanced by straw and biochar applications. The findings indicate that the sole addition of biochar or straw in the current growing season produced better residual positive effects on water conservation, growth promotion, and profit gain in the subsequent growing season in corn-planted saline farmland. In contrast, these effects were more pronounced in the current growing season with the B30 biochar treatment.

## 4 Discussion

In the present study, straw and biochar application generally improved soil moisture content during crop growth periods, especially in the 0-40 cm soil layer in the three-leaf period through tasseling for B30 and BJ (**Figure 2**). The findings are consistent with previous results (Haider et al., 2017; Razzaghi et al., 2020). Returning straw to croplands significantly increases the soil organic carbon content, thereby improving soil total porosity and soil water retention capacity (Cen et al., 2021; Li et al., 2018). The addition of straw to farmland enhances soil aeration and reduces soil compaction and evaporation. The result is the formation of a looser soil structure that is more conducive to irrigation/rainfall infiltration and adsorption (Chen et al., 2022). Feng et al. (2023a) demonstrated that 30 t/hm^2^ of biochar was most effective in suppressing soil evaporation (11%). In addition, straw application creates a favorable environment for microbial growth in the soil, thereby increasing the soil organic carbon content of the soil and improving its water retention capacity (Singh et al., 2023). There are several possible reasons for the enhancement of soil moisture by biochar (Yang et al., 2022; Yuan et al., 2023). Biochar is a hydrophilic material with a porous structure and avidly absorbs water. Addition of biochar to soil improves water retention and storage capacity. However, biochar itself has a large specific surface area, which reduces soil bulk density and increases soil porosity of the amended soil, which is beneficial for increasing soil moisture (Li et al., 2018; Yan et al., 2019). The high salt content of biochar increases soil salinity. This increase in turn increases the moisture absorption capacity, which is beneficial for slowing evaporation of moisture from soil and improving soil moisture environments (Wang et al., 2024). During the three-leaf period, there was no significant difference in the soil moisture content among the treatments. The main reason for this is that the moisture content levels of each treatment were consistent before sowing. The soil moisture content in the top 40 cm soil layer gradually decreased with increasing biochar dosage during the mid-to-late growth period. This may be due to the larger amount of biochar that was applied, which would make the soil more porous and increase the number of soil macropores, which are not conducive to water retention in soil (Razzaghi et al., 2020).

Soil temperature is an important factor that affects crop growth, development, and yield. The average soil temperatures in the top 10 cm soil layer significantly increased upon the addition of straw or biochar (BJ or B30) when averaged over all growing periods each year (**Figure 4**). In contrast, the effect of returning straw to the field on increasing soil temperature was better than the effect of biochar when averaged over the two growing seasons (1.08°C versus 0.97°C). Reducing the surface albedo increases the amount of solar radiation absorbed by the surface, leading to an increased surface temperature (Feng et al., 2021). The straw cover forms a protective layer that prevents fluctuations in soil temperature and maintains soil temperature stability. Biochar is black in color and easily absorbs light of various wavelengths and heat from ultraviolet and visible light (Ding et al., 2019). Moreover, the complex porous structure on the surface of biochar provides a favorable environment for microbial reproduction and survival; many microorganisms release a large amount of heat for reproduction and life activities, resulting in an increase in soil temperature (Obia et al., 2020). The soil surface temperature of each treatment increased with the increase of the amount of carbon applied in the three-leaf stage and jointing stage, because high concentrations of biochar would aggravate the soil color. After the addition of biochar, the decrease of Munsell colorimetric value increased with the increase of the amount of carbon applied, and the Munsell colorimetric value was linearly correlated with the soil albedo. In addition, the plants were smaller in this period, and the differences between different treatments were small, which eliminated the influence of plants on solar radiation. Therefore, the treatment with high concentration of biochar could absorb more solar radiation, and thus increase the soil surface temperature. In addition, the application of biochar increases the soil moisture content, thereby increasing the soil heat flux and reducing the rate of soil temperature decrease. The soil temperatures observed from 8:00 to 20:00 at tasseling, grain-fill, and physiological maturity in the B15 treatment were lower than the temperatures of the corresponding CK (**Figures 3**, **5**). These findings are similar to those reported by Feng et al. (2021). The reduced application of biochar may reduce the surface albedo and increase the amount of solar radiation absorbed by the surface, leading to an increase in surface temperature. In contrast, warming promotes evaporation of moisture from farmland soil, increasing the solar radiation energy consumed by rising water vapor, thus causing a decrease in surface temperature (Chang et al., 2020).

Straw return (BJ) led to a slight reduction in soil pH in the 0-30 cm soil layer during the entire crop growth period in saline-alkali soil (**Figure 6**). Possible cause for this may be because straw return and its decomposition increase the levels of CO_2_ and organic acids in the soil, which may accelerate the leaching of soil cations and lead to a decrease in soil pH (Liang et al., 2023). However, biochar application resulted in a slight increase in the pH during the crop growth period. The pH value of the biochar used in the study was high (pH=9.0), and its mixture into the soil (pH=8.5) could increase the pH of soil-biochar interactions (**Table 1**). This was mainly because of the higher cation exchange capacity after biochar application. The addition of 20 t hm^-2^ biochar reportedly resulted in a cation exchange capacity increase of 17.8% (1.74 cmol_c_ kg^-1^) (Lychuk et al., 2015). With biochar amendment, significant enhancements in pH have been demonstrated and are linked to an increase in CEC (Hailegnaw et al., 2019; Yang et al., 2022). Therefore, the application of biochar to saline-alkali lands may exacerbate soil alkalization. Biochar has no impact on soil pH (Wang et al., 2024) or reduction of pH (Sun et al., 2022). These differences in pH may be related to soil moisture, field management, biochar type, and processing condition of biochars.

Straw BJ and biochar B30 both increased soil salinity throughout the entire growth period of corn during the two growing seasons. This may be because straw and biochar contain more soluble salts or release mineral salts during the decomposition of organic matter. It is also possible that the strong adsorption capacity of biochar promotes the accumulation of salts, leading to increased soil conductivity. In the amendments, soil salts were 1.28~2.83 g/kg at 0-15 and 15-30 cm for each of growing periods (**Figure 6**). Although the total salts displayed increasing trends for the straw and biochar treatments each year, the desalting ratio at the 0-15 and 15-30 cm soil layers from sowing to harvesting in these two amendments was slightly reduced in the first growing season. However, the ratio increased in the 0-30 cm depth soil layer in the second year. The addition of biochar or straw alone in the growing season promoted the downward migration of topsoil salinity, thereby reducing salt accumulation. However, in the second year, in the absence of soil amendments, the topsoil salinity remained relatively high, returning to the initial level before the experiment, and the desalination effect was not significant (**Figure 6**). Therefore, to achieve continuous and uninterrupted reduction of soil salinity, in addition to straw return and biochar, other agricultural management measures (such as irrigation and use of organic manure) may need to be considered. This study considered only one irrigation level, and the changes in salinity were mainly focused on the surface soil. Further research is needed to understand the regulatory effects and mechanisms of straw return and biochar on soil surface and deep-soil salinity under different irrigation methods and amounts.

Positive effects on crop growth, yield, and water productivity have been reported worldwide (Li et al., 2018; Schmidt et al., 2021; Bai et al., 2022; Yang et al., 2024). In this study, as the patterns of soil moisture, heat, and salt showed an incompletely consistent response to straw or biochar (**Figures 2**-**6**), crop dry biomass, yield, WUE, and net profit were all enhanced by straw or biochar in the current and subsequent crop growing seasons (**Figure 7**). These effects improved more during the first growing season than during the second. This may be because fewer rainfall events were recorded during the second year (**Figure 1**). These findings indicate that these two amendments had positive residual effects on crop productivity. The applications of biochar and straw mulching effectively reduced soil water consumption throughout the growth period, thereby improving the WUE. Biochar contains a large amount of organic matter, which allows crops to fully absorb and utilize the nutrients released during their growth and subsequent development. Yang et al. (2023) demonstrated that, based on these improvements in soil organic matter, available nutrients, fertilizer productivity, and economic benefits, biochar amendment at 30 t ha^-1^ is a suitable rate for three years in a corn cropping system with drip irrigation in the same temperate zones. Simultaneously, the porous structure of biochar can increase the soil microbial biomass and community structure, thereby accelerating the decomposition of organic matter in the soil, improving soil nutrients, and providing sufficient nutrients for various plant growth stages (Hu et al., 2024). Straw return or biochar improves the soil aggregate structure of the cultivated layer in arid areas (Bo et al., 2023), effectively enhances soil water storage and retention capacity during the fallow and growth periods, and significantly improves crop yield and water use efficiency. In contrast, we observed that WUE and net profit in B30 increased more than those in BJ when averaged over two years. Thus, a biochar rate of 30 t ha^-1^ is recommended for better soil water conservation, heat storage, crop yield, WUE improvement, and net income enhancement without a significant increase in the degree of soil salinization. Due to the high cost of biochar production and field application, this study only considered the water-saving, salt-controlling and yield-increasing effects of corn fields with biochar gradients of 15 and 30 t ha^-1^, whether there is a more suitable amount of straw biochar, the long-term field test (Haider et al., 2017; Cong et al., 2023) and biochar model simulations (Jiang et al., 2023) should be considered under human activities.

## 7 Conclusion

A 2-year field experiment were conducted to systematically compare the response of soil water, temperature, and salinity and crop water use efficiency to the one-time application of straw and biochar in saline maize farmland. Both straw direct return to the field and straw biochar can increase the soil moisture content and soil temperature cm in the later stage of crop growth in the first and second growing seasons. Compared with straw direct return, high dosage of biochar has a better water retention and holding effect, while its insulation effect is not as good as straw direct return to the field. The diurnal observation periods for improving the soil temperature were most concentrated between 08:00-12:00 for BJ and 10:00-2:00 for B30.

The application of biochar did not significantly increased soil salinity and pH value during the crop growth period, while the direct return of straw significantly reduced the soil salinity during the second year of crop growth while it did not change pH significantly. Straw return and biochar also enhanced crop yield, WUE, net income, and reduced actual evaporation every year, and these effects improved more in the first growing season than in the second growing season. B30 (30 t ha^-1^) is suitable for improving soil water and heat storage, crop WUE, and benefit without increasing the degree of soil salinization.

## Data Availability

The original contributions presented in the study are included in the article/supplementary material. Further inquiries can be directed to the corresponding author.
